# Spatial visualization provides insight into immune modulation by an L-DBF vaccine formulation against *Shigella*

**DOI:** 10.3389/fimmu.2025.1577040

**Published:** 2025-04-23

**Authors:** Ti Lu, Skyler T. Kramer, Mary A. York, Mst Nusrat Zahan, Debaki R. Howlader, Zackary K. Dietz, Sean K. Whittier, Nathan J. Bivens, Alexander Jurkevich, Lyndon M. Coghill, William D. Picking, Wendy L. Picking

**Affiliations:** ^1^ Bond Life Sciences Center and Department of Veterinary Pathobiology, University of Missouri, Columbia, MO, United States; ^2^ Bioinformatics and Analytic Core, University of Missouri, Columbia, MO, United States; ^3^ Genomics Technology Core, University of Missouri, Columbia, MO, United States; ^4^ Advanced Light Microscopy Core, University of Missouri, Columbia, MO, United States

**Keywords:** *Shigella*, intranasal vaccine, spatial transcriptomics, immune modulation, fibroblasts, cardiomyocytes

## Abstract

Shigellosis remains a global public health problem, especially in regions with poor sanitation measures. Our prior work has demonstrated the protective efficacy of a three-dose regimen of L-DBF, a recombinant fusion of IpaD and IpaB from *Shigella flexneri* with the LTA1 moiety of enterotoxigenic *E. coli* labile toxin. Here, we investigate how a two-dose regimen (one prime and one booster) of L-DBF, formulated in an oil-in-water emulsion called ME, modulates immune responses in the lung using a spatial transcriptomics approach. Our findings show significant changes in the lung immune landscape following the vaccination, including increased expression of B cell markers, antigen presentation genes, and T cell-associated markers. Our analysis also revealed significant reprogramming of fibroblasts and cardiomyocytes, showing that fibroblasts are shifted from extracellular matrix production to immune modulation, while cardiomyocytes enhanced the signaling for immune cell recruitment and vascular stability. The communication between alveolar type 2 (AT2) cells and cardiomyocytes also increased, reflecting coordinated support for immune readiness and maintaining tissue integrity. These findings underscore the potential of L-DBF/ME vaccination to enhance both humoral and cellular immunity, as well as to reshape lung immune architecture while enhancing immune readiness, thereby offering a promising approach for effective protection against *Shigella* infections.

## Introduction

*Shigella* is a human-adapted intracellular pathogen that causes severe diarrhea and dysentery, leading to an estimated 270 million infections and 212,000 deaths worldwide annually (1). The genus includes *S. dysenteriae*, *S. flexneri*, *S. boydii*, and *S. sonnei*, which are classified into over 50 serotypes based on O-antigen structure (1). *S. flexneri* is particularly prevalent in low- and middle-income countries, posing a significant public health challenge (2). Despite the substantial global burden of *Shigella* infections, no licensed vaccine is available (3). Developing an effective vaccine against *Shigella* is crucial for improving global health, especially in vulnerable populations (4).

A key element of *Shigella* pathogenicity is its type III secretion system (T3SS, see **Supplementary Table S1**), which delivers virulence factors into host cells to evade immune detection and establish infection (5). Two crucial components of the T3SS, the needle tip protein IpaD and the translocator protein IpaB, are highly conserved across all *Shigella* serotypes, making them promising targets for a serotype-independent subunit vaccine (1, 5). Our previous studies have shown that intranasal administration of IpaD and IpaB (6), or the IpaD-IpaB fusion (DBF) (7), combined with the mucosal adjuvant dmLT (double mutant heat-labile enterotoxin from enterotoxigenic *E. coli*), provides cross-protection against both *S. flexneri* and *S. sonnei*. To simplify vaccine production, a recombinant fusion protein, L-DBF, was developed, which integrates LTA-1 (the active subunit of dmLT) with IpaB and IpaD (8). In multiple formulations, this fusion protein has demonstrated broad-spectrum protection against lethal *Shigella* challenges using a mouse pulmonary infection model (4, 9). While this model is not ideal, it has provided a starting point for vaccine development against this human-adapted pathogen. Both Th17 and Th1 cells were found to be important in defense against *Shigella* infection, with Th17 cells promoting pathogen clearance through IL-17 mediated processes and Th1 cells activating macrophages via IFN-γ to control intracellular replication (9, 10). However, there remains a gap in understanding how these immune responses are coordinated within distinct lung regions and microenvironments following intranasal administration of L-DBF.

For intranasal vaccines to target gut pathogens like *Shigella*, it is essential to investigate the local tissue-specific immune responses, which is also true for the lung infection model. This is particularly true for those responses involving non-traditional immune cells and tissue-resident populations (11). The respiratory mucosa is a model site for exploring immune activation across the entire host mucosal system. Here we plan to first understand how lung-based immune responses are modulated by our vaccine formulation so that this can later be correlated with enhances protection at other mucosal regions, such as the gastrointestinal tract (11, 12). This knowledge is vital for optimizing host defenses against *Shigella* infection. Toward this end, spatial transcriptomics technology (10X Visium) offers a powerful tool to map gene expression in specific tissue regions, providing valuable insights into how immune responses are organized locally (13). By revealing how immune cells interact and function within distinct lung microenvironments following vaccination, spatial transcriptomics can inform the design of more effective intranasal vaccines (14). This approach helps optimize immune activation in the respiratory tract, enhancing mucosal immunity and potentially preventing the spread of pathogens like *Shigella* that establish infection in the gut (15).

Here, we applied spatial transcriptomics to analyze transcriptomic changes in mouse lung cells after intranasally administering a two-dose regimen (one prime and one booster) of L-DBF as part of a squalene-based oil-in-water emulsion formulation called ME (MedImmune Emulstion) and evaluated its protective efficacy against an otherwise lethal *Shigella* challenge in a mouse respiratory model (1, 4, 16). Following vaccination, we analyzed the diverse cell types and immune responses throughout the lung, focusing on functional changes in non-immune cells, such as fibroblasts and cardiomyocytes. Fibroblasts and cardiomyocytes, traditionally structural cells in lungs, have been found to contribute to immune regulation via multiple pathways (17, 18). By examining signaling pathways related to extracellular matrix (ECM) interactions, immune cell recruitment, and tissue repair, we aimed to reveal the dual roles of these cells in maintaining structural integrity and modulating immune responses during vaccination. This study provides initial valuable insights into how vaccination influences the behavior of non-immune cells to support lung defense, ensuring the tissue remains both structurally resilient and immunologically prepared for future infections. These findings have broader implications for understanding tissue-level changes induced by vaccination and may inform strategies to improve vaccine efficacy while reducing tissue-related side effects.

## Materials and methods

### Materials

Squalene was purchased from Echelon Biosciences (Salt Lake City, UT). Chromatography columns were from GE Healthcare (Piscataway, NJ). All other reagents were from Sigma or Fisher Scientific and were chemical grade or higher.

### Protein preparation

IpaD, IpaB, and L-DBF were made as previously described (6–8). Briefly, IpaD, IpaB, and L-DBF were produced in *E. coli* Tuner(DE3) cells using IPTG (1 mM) to induce expression. After 3 h, cells were lysed, the supernatant clarified by centrifugation, and the proteins purified using standard IMAC (Immobilized metal affinity chromatography; Cytiva, Marlborough, MA) followed by Q anion exchange chromatography to remove LPS. IpaD was dialyzed into a buffer containing 20 mM histidine (pH 7.4), 100 mM NaCl, and 5% sucrose, and frozen at -80°C. IpaB and L-DBF were co-expressed with the IpaB chaperone IpgC, which had an N-terminal His_6_ tag. The IpgC was separated from IpaB and L-DBF by adding 0.05% lauryl-dimethylamine oxide (LDAO) and then removed via a second IMAC step. IpaB and L-DBF were stored in histidine buffer (20 mM histidine, pH 6.5), containing 100 mM NaCl, 5% sucrose, and 0.05% LDAO, at -80°C. Endotoxin levels were confirmed to be <5 EU/mg protein using EndoSafe analysis (Charles River Laboratories, Wilmington, MA).

### Preparation of L-DBF + ME formulations

Squalene (8% by weight) and polysorbate 80 (2% by weight) were mixed to achieve a homogenous oil phase as previously described (19). A histidine buffer (40 mM histidine, pH 6) with 20% sucrose was added to the oil phase using a Silverson L5M-A high-speed mixer at 7,500 RPM. The mixture underwent six passes in a Microfluidics 110P microfluidizer at 20,000 psi to generate a 4X emulsion (ME). The antigen was admixed with ME, which was then adjusted to 1X prior to vaccination.

### Mice and immunizations

**Figure 1:**
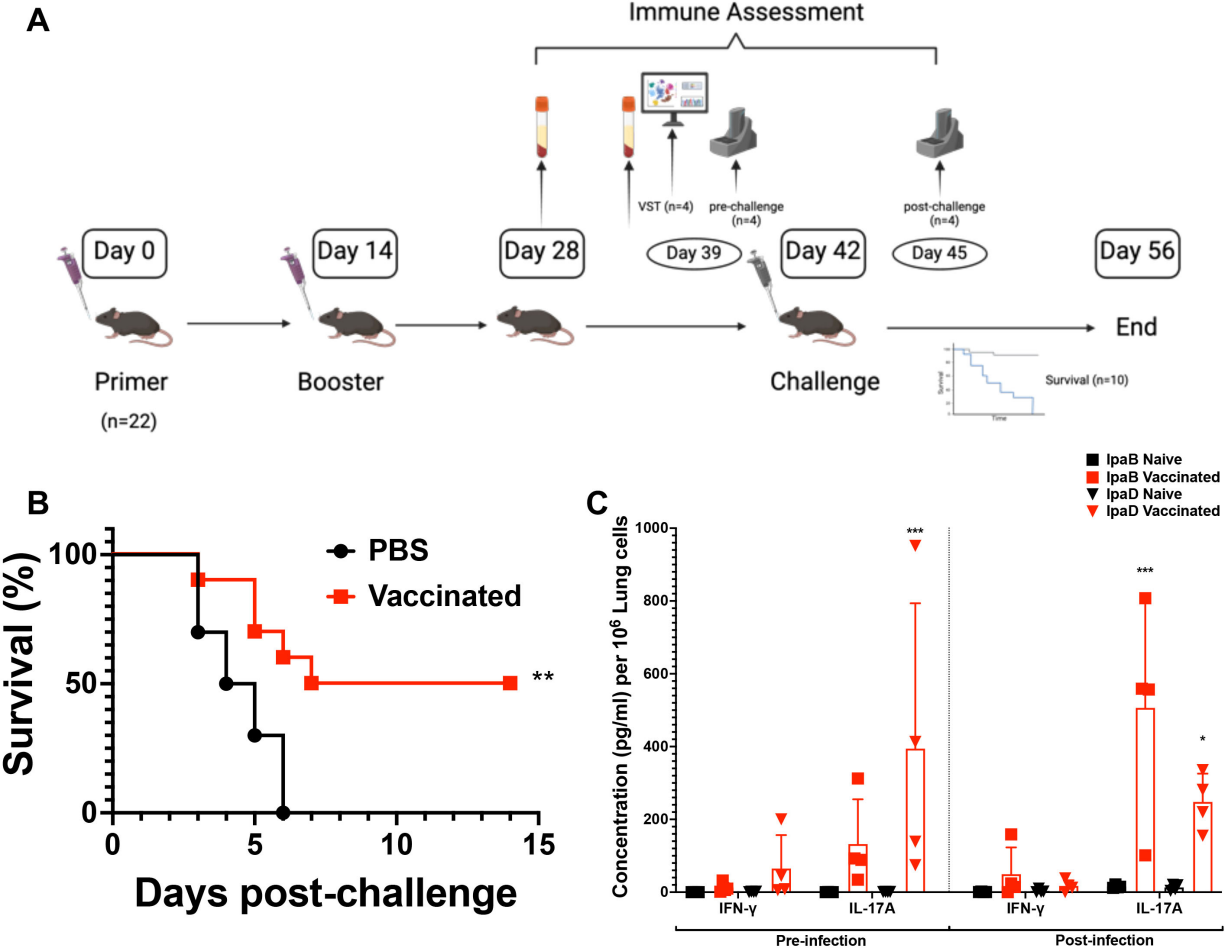
The experimental workflow of vaccination and the efficacy of two doses of L-DBF/ME in C57BL/6 mice. **(A)** In the experimental setup, mice were vaccinated with PBS (placebo or naïve group) or 10 μg L-DBF formulated with ME intranasally, and subsequently challenged with *Shigella flexneri* 28 days after the final vaccination (Figure created using ). Notably, the data presented in this paper were all conducted within a single animal study. **(B)** Survival following challenge with *S. flexneri* is shown with the percentage of survival over 14 days post-challenge presented for naïve (black line) and vaccinated (red line) mice. Significance was calculated by using Log-rank (Mantel-Cox) test with **p=0.005. **(C)** Cytokine levels from pre- and post-infection in naïve (black) and vaccinated (red) groups were determined by Meso Scale Discovery analysis as per the manufacturer’s specifications. They are presented here as pg/ml/10^6^ lung cells. Secretion of different cytokines was noted as a response to either IpaB or IpaD stimulation. Data were plotted as actual values from individuals ± SD (n = 4) in each group. Significance was calculated by comparing groups that were unvaccinated (PBS) and mice vaccinated with antigens using a Welch t-test. *p<0.05; ***p< 0.001.

Mouse experiments were approved by the Office of Animal Research (OAR) and the institutional IACUC at the University of Missouri (Protocol: 38241). Six-to-eight-week-old female C57BL/6 mice (Charles River Laboratories, Wilmington, MA) were used in this study. For intranasal administration, 30 µL of either PBS, pH 7.4 (placebo) or 10 µg L-DBF/ME was prepared. Mice were anesthetized using isoflurane and vaccine formulations administered intranasally (IN) as previously described (4, 9). Vaccinations were conducted on Days 0 and 14 (prime and boost, respectively), with groups of 22 mice divided as follows: 10 for survival, 4 for pre-challenge immune assessment, 4 for post-challenge immune assessment, and 4 for spatial transcriptomic sample collection (**Figure 1A**). Notably, the survival curve, antibody titers, cytokine analysis, and spatial transcriptomics were all conducted within a single animal study.

### Antibody ELISAs

Blood was collected for serum preparation on Days 27 and 42. Anti-IpaD and -IpaB IgG titers were measured as previously described (9). Microtiter wells were coated with 100 ng IpaB or IpaD in PBS, incubated at 37°C for 3 hours, and blocked overnight with 10% nonfat dry milk in PBS. Sera were added as the primary antibody for 2 h, followed by an HRP-secondary IgG antibody (1:1000) for 1 hour. After washing, OPD (o-phenylenediamine dihydrochloride) substrate was added, and detection was done at 490 nm via ELISA (enzyme-linked immunosorbent assay). Endpoint titers were calculated and represented as ELISA units per ml (EU ml^-1^).

### *Shigella* challenge studies

*S. flexneri* 2a 2457T challenge strains were grown on tryptic soy agar containing 0.025% Congo red at 37°C, subcultured in tryptic soy broth to an A600 of 1.0, and diluted to 1 x 10^6^ CFU in 30 µl PBS for intranasal (IN) challenge. Mice were monitored for two weeks, with euthanasia criteria including >25% weight loss or blood glucose ≤100 mg/dL. Remaining mice were euthanized on Day 14 post-infection (9). Mice were euthanized using carbon dioxide (CO_2_) inhalation with a flow rate of 10-30% volume/min and following IACUC guidelines. The process involved gradually filling the chamber with CO_2_ to minimize distress, followed by confirmation of death based on standard protocols.

### Cytokine determinations

Lung cells were collected on day 53 (three days before the remaining mice were challenged) and incubated with 10 µg/ml IpaB, IpaD or PBS for 48 h at 37°C, as previously described (9). Supernatants were analyzed using U-PLEX kits for IFN-γ and IL-17A cytokines. Concentrations were measured via MSD plate reader and associated software (Meso Scale Discovery, Rockville, MD).

### Sample preparation for mouse lung for spatial transcriptomics

To prepare for tissue collection, isopentane was chilled in a metal beaker placed on dry ice to ensure rapid tissue freezing. Lung samples were collected from both naïve and vaccinated mice (n=4 per group) based on a published protocol (20). Mice were sacrificed, and a laparotomy was performed to expose the thoracic cavity. Lungs were perfused via a tracheal needle with an Optimal Cutting Temperature (OCT)/PBS solution (1:1) (20). After inflation, extracted lungs were rinsed in OCT, coated with room-temperature OCT in a pre-cooled cryomold, and placed in chilled isopentane until OCT solidified and turned white (20). Samples were stored at -80°C for subsequent cryo-sectioning and analysis.

### Tissue sectioning, staining and imaging

Tissue samples were cut using a Leica CM3050 S cryostat (Leica Biosystems, Deer Park, IL), and 10 µm thick sections were placed on capture areas of Visium spatial gene expression slides v1 (10X Genomics, Pleasanton, CA), as described previously (21). Slides were fixed in pre-chilled methanol (-20°C) for 30 minutes and stained with Mayer’s hematoxylin for 7 minutes. Sections were washed in nuclease-free water, incubated in bluing buffer (Dako) for 2 minutes, counterstained with eosin for 1 minute, briefly rinsed, and dried on a slide warmer at 37°C for 5 minutes. Images of individual capture areas with stained sections were acquired using a Zeiss Axiovert 200M motorized microscope (Carl Zeiss Microscopy, White Plains, NY) with a 5x Plan Apochromat objective and a Leica DFC290 camera. Image tiles were stitched using Metamorph software (Molecular Devices, LLC, San Jose, CA). The pixel size of the final composite images was 1.275 µm.

### Permeabilization, reverse transcription, second strand synthesis, and cDNA PCR amplification

The optimal enzymatic permeabilization time for the tissue type was pre-determined using the 10X Genomics Tissue Optimization kit, as described previously (21). The stained and imaged slides were incubated in the permeabilization enzyme solution for 8 min at 37˚C. Following the permeabilization wash step, the reverse transcription master mixture was added and incubated for 45 min at 53˚C. After degradation of the mRNA with KOH, the second strand synthesis was carried out for 15 minutes at 65˚C by adding a second strand mixture containing the second strand enzyme and primer. The second strand cDNA was released by denaturation of the double-stranded cDNA with KOH, neutralized with 1 M Tris-HCl and transferred from the slide section to an 8-tube strip. After determinating the optimal PCR cycle number by KAPA SYBR FAST qPCR Master Kit, the cDNA was amplified for 15 cycles using the PCR cycling conditions provided in the 10X Genomics Visium Spatial Gene Expression protocol. The cDNA was purified using 0.6X AxyPrep Mag magnetic beads. The quality and concentration were confirmed by the Fragment Analyzer (Agilent) using the High Sensitivity NGS kit.

### Spatial gene expression library construction

The cDNA library for the Illumina platform was prepared by following the 10X Genomics spatial gene expression library construction protocol, as described previously (21). Briefly, the double-stranded cDNA was fragmented, the end was repaired, and an A-tail was added in a single step. The A-tailed fragments were size selected using double-sided AxyPrep Mag magnetic beads with a bead-to-reaction solution ratio of 0.6X for the first step and 0.8X for the second step. The adaptor was ligated and purified with 0.8X AxyPrep Mag magnetic beads. The final Illumina libraries were amplified by PCR, which added the sample dual indexes. The PCR amplified libraries were purified and size selected using double-sided AxyPrep Mag magnetic beads with a bead-to-reaction solution ratio of 0.6X for the first step and 0.8X for the second step. The individual library was analyzed by Fragment Analyzer (Agilent) to determine the quality and average fragment size and quantified using the Qubit (Invitrogen) DNA assay. The libraries were pooled in equal molar ratios with a total concentration of 5 nM and sequenced on the Illumina Novaseq 6000.

### VST analysis

The analysis of spatially resolved RNA-seq data was completed in R v4.4.1 (22) with Seurat v5.1.0 (23). The VST data were clustered on a per-spot basis. In brief, these samples were merged, normalized via the SCTransform method (24), processed via PCA, and then clustered according to the default Seurat method (resolution = 0.1). Positive cluster markers were identified after filtering genes according to the following criteria: the minimum log_2_ fold-change between populations must be at least 0.5, and at least 50% of the spots in either population must express the gene of interest. For each cluster, differentially expressed genes between the naive and vaccinated conditions were identified after filtering the genes with the same fold-change threshold and a population threshold of 5%, providing a more comprehensive view of gene expression patterns in these two groups.

### Cell communication analysis

The communication analysis was completed in R v4.4.1 (22) with CellChat v2.1.2 (25), using the mouse database of ligand-receptor pairs. Default package functions were used to identify over-expressed genes/interactions, compute the communication probability for signaling pathways, and perform differential communication analysis.

### Statistical analyses

GraphPad Prism 8.1.2 was used to prepare data and perform statistical analyses. Using Dunnett’s multiple comparison test, PBS groups were compared with the other vaccinated groups. A p-value below 0.05 was considered significant (*p < 0.05, **p < 0.01, ***p < 0.001).

## Results

### Two-dose regimen (one prime and one boost) of the intranasal L-DBF/ME formulation elicits moderate protection and potent Th17 immune responses in C57BL/6 mice against lethal *Shigella* challenge

Our previous studies showed that L-DBF in an oil-in-water emulsion called ME (L-DBF/ME) admixed with the TLR-4 agonist and lipid A analogues BECC438 or BECC470 could induce effective protection against *Shigella* spp. infections in young BALB/C mice (4). To simplify the formulation, we assessed the immune response elicited by a two-dose intranasal regimen (one prime and one boost) of 10 µg L-DBF/ME without BECC before and after a lethal *Shigella* challenge in female C57BL/6 mice, a strain that provides a more genetically relevant background for studying *Shigella* infections and immune responses due to its broader use in immunological research (6-8 weeks; n=22, 10 for survival, 4 for pre-infection necropsy, 4 for post-infection, 4 for VST; all data were all conducted within a single animal study.) (**Figure 1A**) (26). Half of the C57BL/6 young mice vaccinated with 2 doses of 10 µg L-DBF/ME survived the challenge (1 × 10^6^ CFU/mouse), while all mice in the PBS group rapidly succumbed to the challenge (*p*=0.005; **Figure 1B**, **Supplementary Figure S1A**).

We next evaluated the immune responses for identically treated mice prior to challenge to identify differences in antibodies and cytokine secretion induced by the L-DBF/ME formulation. Elevated anti-IpaD and -IpaB IgG were observed in response to vaccination (**Supplementary Figure S1B**). Furthermore, elevated secretion of IL-17 was seen in the lungs before and after *Shigella* infection (**Figure 1C**). This cytokine response is indicative of a potent Th17 immune responses after only two doses of the vaccine, thereby suggesting an effective cellular immunity and controlled inflammation.

### The spatial expression map displays the higher expression of key gene markers in vaccinated mice compared to naïve mice​​

**Figure 2:**
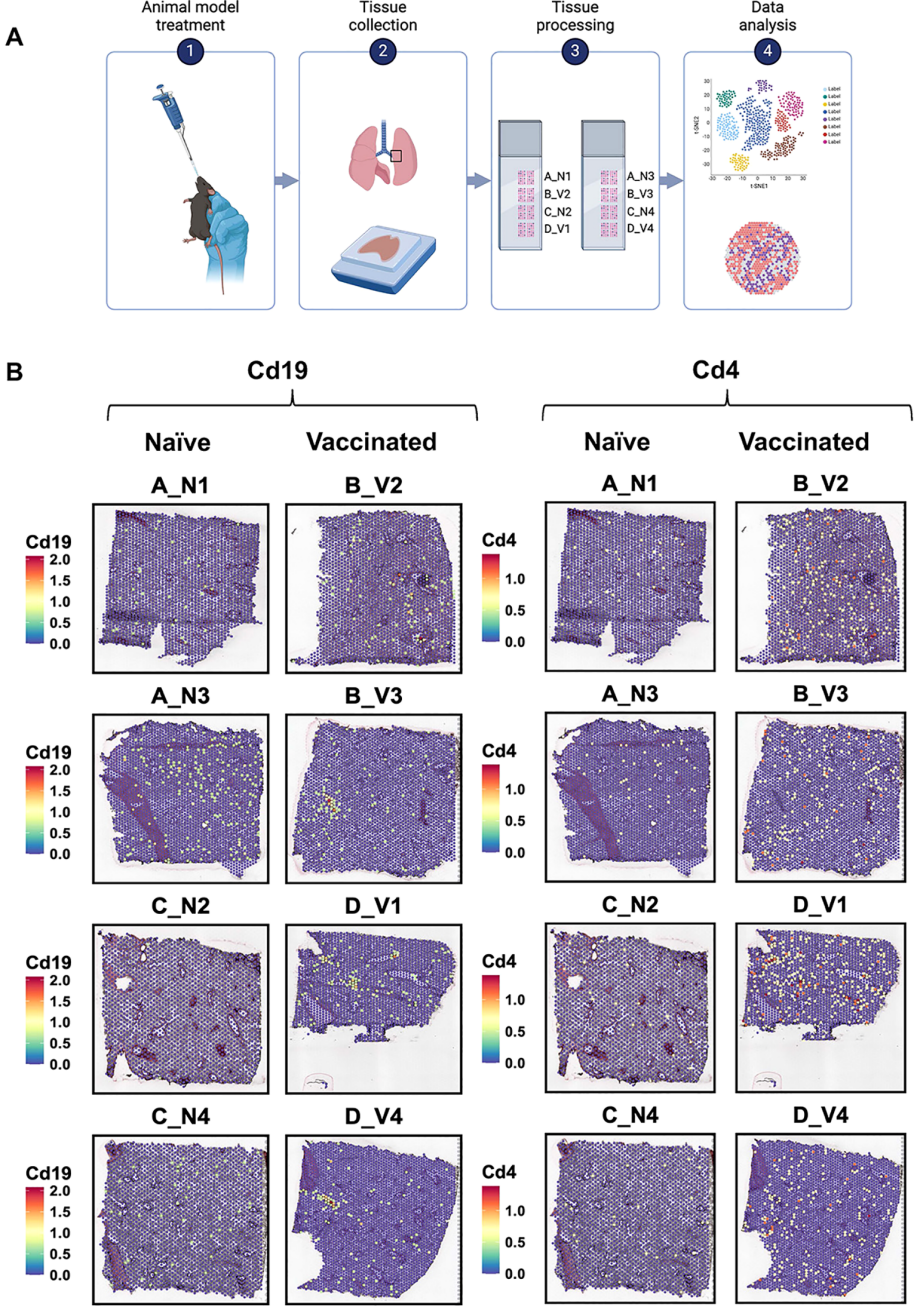
The experimental workflow of spatial transcriptomic and gene expression analysis of lung tissues from naïve and vaccinated mice. **(A)** The spatial transcriptomic experiment includes four steps: intranasal vaccine administration, lung extraction, tissue processing for spatial transcriptomics, and data analysis. (Figure created using ) **(B)** Spatial transcriptomic maps of lung sections, showing the distribution and expression levels of the markers *Cd19* (left) and *Cd4* (right). Each panel represents a different sample, labeled as A_N1, B_V2, A_N3, B_V3, C_N2, D_V1, C_N4, and D_V4. The color scale on each map indicates the expression level of the markers, with red and yellow denote higher expression levels, while blue represents lower levels. The labels “N” and “V” suggest naïve and vaccinated groups, respectively, with numbers indicating specific sample identifiers.

Using the 10x Genomics Visium spatial gene expression platform, we generated spatial transcriptomes for lung sections from naïve and vaccinated mice (n=4, **Figure 2A**). Barcoded spots for 10-μm thick sections were printed on the capture areas (6.5 mm × 6.5 mm) in Visium slides (21). From these spots, we initially identified the spatial expression maps which revealed significant differences in the location and intensity of gene expression between the naive and vaccinated groups. We focused on markers such as B-cell markers (*Cd19*, *Cd79a*; **Figure 2B** Left, **Supplementary Figure S2**), T-cell markers (*Cd4*, *Cd3e*, *Cd8a*; **Figure 2B** Right, **Supplementary Figure S3**), macrophage markers (*Fcgr1*) (27), eosinophil markers (*Prg2*) (28), and pro-inflammatory cytokine markers (*Tnf*) (29) (**Supplementary Figures S4**, **S5**). In the naïve (placebo) group, the expression of each focused marker was minimal and dispersed, suggesting a baseline, non-activated state of immune cells throughout the tissue.

In the vaccinated group, localized increases in *Cd19*, *Cd3e*, *Cd4*, *Cd79a*, *Cd8a*, *Fcgr1*, *Tnf* and *Prg2* expression were observed, particularly in samples B_V3 and D_V1 (**Figure 2B**, **Supplementary Figures S2**-**S5**). We observed a similar spatial distribution trend for B cell-related markers (*Cd19*, *Cd79a*) across the lung samples of all vaccinated mice. Those patterns, however, differed from the distribution of T cell-related markers (*Cd4*, *Cd3e*, *Cd8a*). Increased expression of these immune related markers within regions suggested there is localized immune activation in vaccinated lungs.

### Distinct cell clusters characterized by unique gene expression profiles are seen within the mouse lung after vaccination

**Figure 3:**
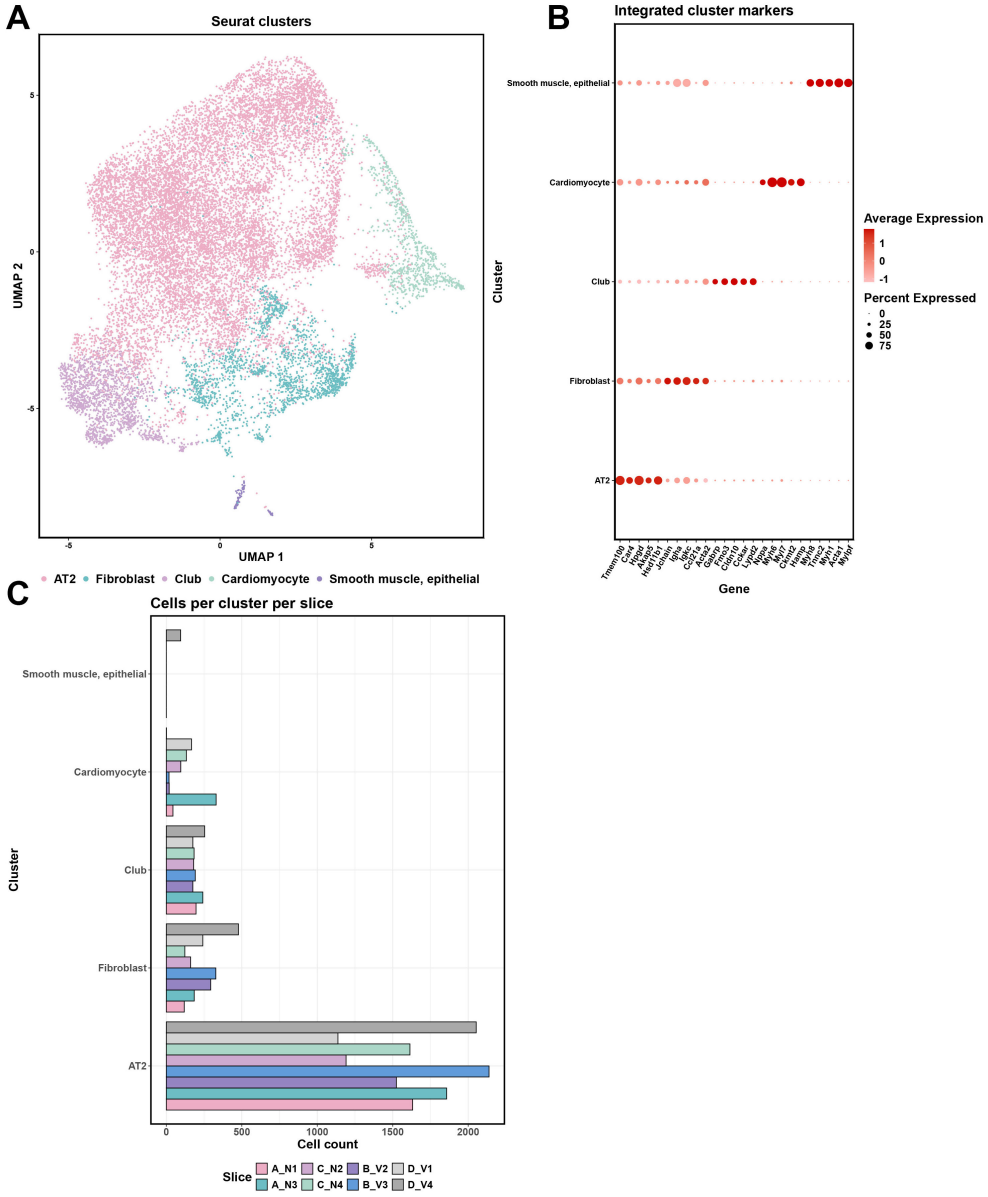
Comprehensive cellular clustering and gene expression analysis of the spatial transcriptomics data. **(A)** UMAP plot of Seurat clusters, color-coded to represent different clusters (Alveolar type 2 (AT2) cells; Fibroblasts; Club cells; Cardiomyocytes; Smooth Muscle and Epithelial cells) identified within the tissue samples. **(B)** Integrated cluster markers with gene expression levels for each cluster. Dot size represents the percentage of cells expressing the gene, and color intensity reflects the average expression level. **(C)** Distribution of cells across clusters for each sample (A_N1, B_V2, A_N3, B_V3, C_N2, D_V1, C_N4, and D_V4), providing a count of cells per cluster per tissue slice.

We quantified the marker genes for cell clusters based on log_2_ fold-change, selecting the top 10 markers from each cluster for further analysis. These selected markers were then used to annotate the clusters by referencing the Mouse Cell Atlas (https://bis.zju.edu.cn/MCA/). This annotation process identified five major cell types across the clusters (**Figure 3A**): Cluster 0- Alveolar type 2 (AT2) Cells; Cluster 1-Fibroblasts; Cluster 2-Club cells; Cluster 3-Cardiomyocytes; Cluster 4-Smooth Muscle and Epithelial cells (**Figure 3B**, **Supplementary Figure S6A**). Notedly, the detection of cardiomyocytes likely reflects their anatomical proximity to the pulmonary veins and bronchi (30). While Cluster 4 was exclusively identified in section D_V4, our analysis in this study primarily focused on Clusters 0-3 (**Figure 3C**, **Supplementary Figure S6B**).

**Figure 4:**
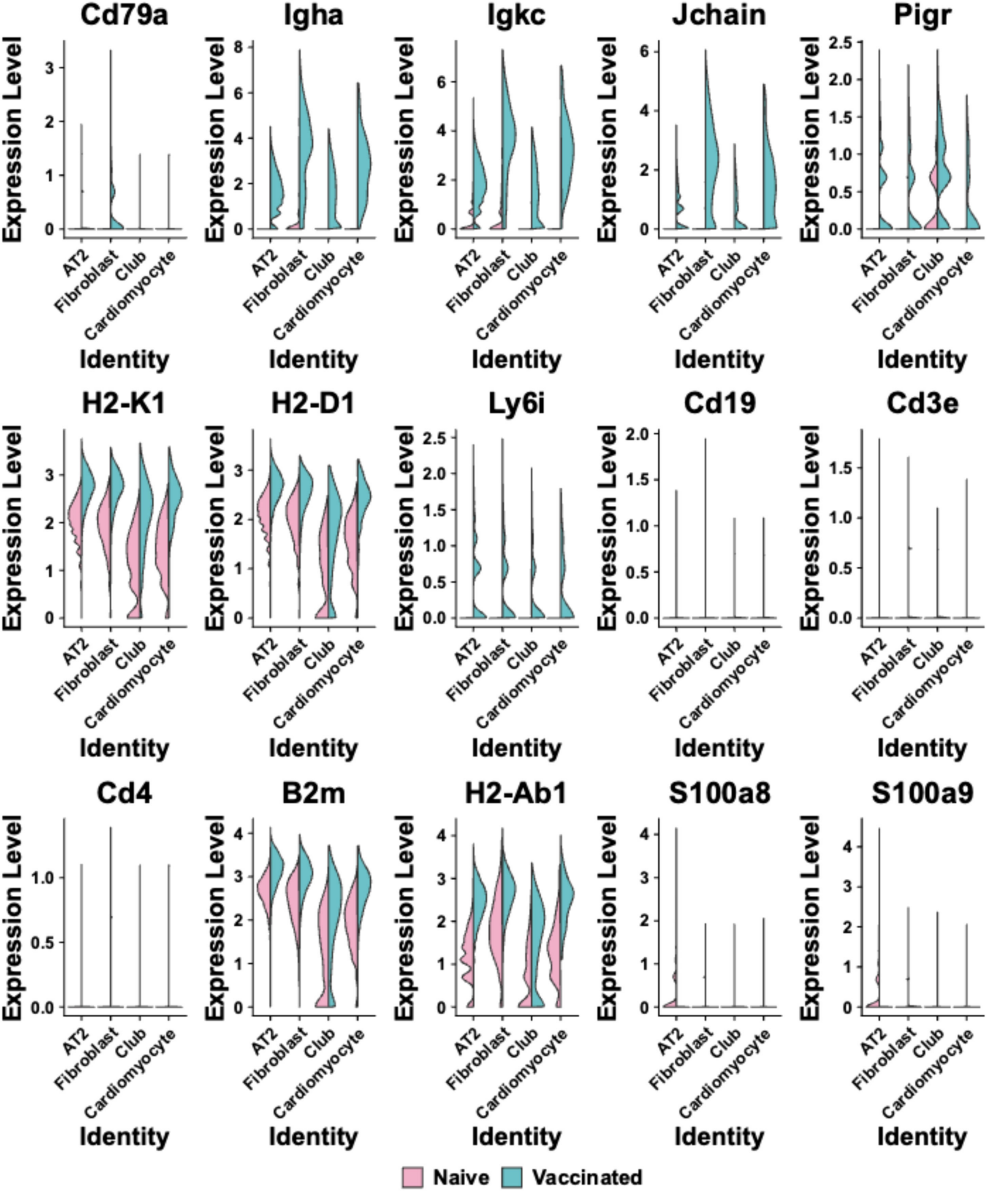
Violin plots are shown comparing the expression levels of selected immune-related genes across different cell clusters (Alveolar type 2 (AT2) cells; Fibroblasts; Club cells; Cardiomyocytes) identities in naïve and vaccinated groups. Each plot represents a different gene, including *Cd79a*, *Igha*, *Igkc*, *Jchain*, *Pigr*, *H2-K1*, *H2-D1*, *Ly6i*, *Cd19*, *Cd3e*, *Cd4*, *B2m*, *H2-Ab1*, *S100a8*, and *S100a9*. The x-axis labels cell cluster identities, and the y-axis represents expression levels. Pink represents the naïve group, while blue indicates the vaccinated group.

Across all spots corresponding to the clusters 0-3, there was a generally higher expression of genes related to B-cell and T-cell activation and antigen presentation (**Figure 4**). The expression levels of *Igha*, *Igkc*, *Jchain*, and *Pigr* were notably higher and more widespread in vaccinated mice across these cluster-specific spots (Cluster 0-3: *p* < 0.05, **Figure 4** Blue), whereas in naïve samples, these genes exhibited lower expression and were confined to specific clusters (**Figure 4** Red). The genes *H2-K1, H2-D1, H2-Ab1*, and *B2m* showed broad expression across all cluster-specific spots in both naïve and vaccinated groups, but the expression levels were significantly higher in vaccinated mice (Cluster 0-3: *p* < 0.05, **Figure 4**). The expression levels of B-cells related markers *Cd19* and *Cd79a* were higher and showed wider spatial distribution across lung tissue sections in vaccinated mice, particularly in spots within Cluster 1 and 3 (**Supplementary Table S2**, **Figure 4**). T-cell markers like *Cd3e* (Cluster 0-3), *Cd4* (Cluster 0, 1, 3), *Cd8a* (Cluster 1) showed modestly higher expression in vaccinated groups across these cluster-specific spots (Naïve vs. Vaccinated: *p*<0.05 with an average log_2_ fold-change of -2.7), suggesting a detectable T-cell mediated response, which is critical for effective adaptive immunity (**Supplementary Table S2**, **Figure 4**).

Interestingly, the *Ly6i* expression was found to be moderately increased across all spots corresponding to the four clusters in the vaccinated group compared to the naïve group (**Figure 4**), indicating enhanced activation or priming of T-cells and myeloid cells in response to vaccination. In contrast, the expression levels of *S100a8* and *S100a9* following vaccination showed either a minimal increase or small reduction in most cluster-specific spots (notably in Cluster 0; Naïve vs. Vaccinated: *p*<0.05 in cluster 0-3 with an average log_2_ fold-change of 1.4), compared to their expression levels in naïve mice. These two markers are involved in inflammatory responses, specifically associated with neutrophils (31, 32). Lack of their significant upregulation in the lung of vaccinated mice suggests that the vaccination did not induce inflammation or neutrophil activation at 28 days after the last vaccination (**Supplementary Table S2**, **Figure 4**). This comprehensive expression profile in cluster-specific spots underscores the capacity of intranasal L-DBF/ME to enhance both humoral and cellular immunity, which are both pivotal for protecting against *Shigella* infection.

### The changes in cell communication among different cell clusters post-vaccination

**Figure 5:**
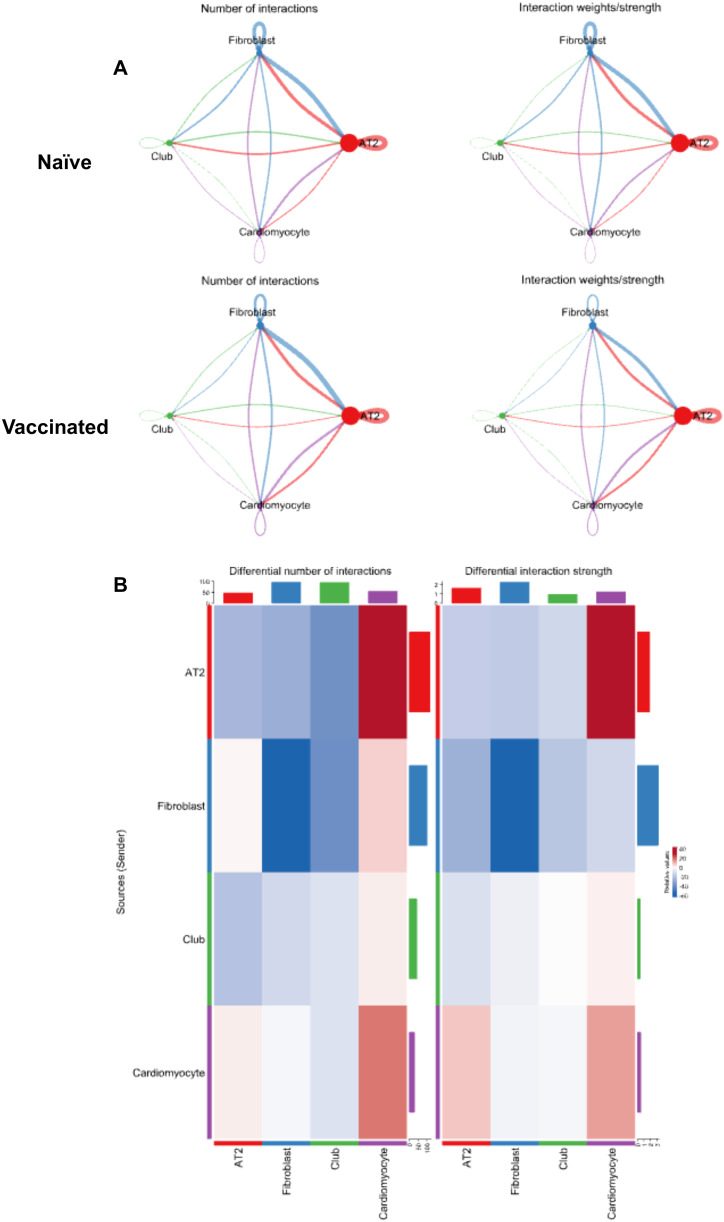
A network analysis of cellular interactions and their dynamics in response to treatment. **(A)** Network diagrams illustrating the number of interactions (left) and interaction weights/strengths (right) among different cell clusters (Alveolar type 2 (AT2) cells; Fibroblasts; Club cells; Cardiomyocytes) in both naïve and vaccinated groups. The lines connecting clusters vary in color and thickness, indicating the interaction strength and frequency. **(B)** Heatmaps of the differential number of interactions (left) and interaction strength (right) between clusters in vaccinated versus naïve groups, with color intensity representing the degree of change and bars indicating specific values.

To delve deeper into the changes in cell communication among different cell clusters following the intranasal vaccination of L-DBF/ME, we used CellChat to visualize the aggregated cell-cell communication network between specific clusters (AT2 cells, Fibroblasts, Club cells, and Cardiomyocytes) (25). The interactions between most of the clusters remained stable, with no substantial changes in connection strength or patterns (**Figure 5A**), however, the most prominent shift observed post-vaccination was the increased communication between AT2 cells (Cluster 0) and cardiomyocytes (Cluster 3). In contrast, a detectable reduction in interaction strength was noted between fibroblasts (Cluster 1) and club cells (Cluster 2), as well as between AT2 cells (Cluster 0) and club cells (Cluster 2).

Analysis of the differential communication heatmap generated from the VST provided a comprehensive insight into the shifts in cellular communication within lung tissue post-vaccination (**Figure 5B**). The decrease in both the number (**Figure 5B** Left) and strength (**Figure 5B** Right) of fibroblast self-interactions (Cluster 01 to 01) indicated that fibroblasts were less engaged in repair processes within the lung tissue post-vaccination. This suggests that the vaccine enhanced the stabilization of the lung tissue, reducing inflammation and tissue damage, minimizing the need for fibroblast-driven repair and ECM (extracellular matrix) production.

The increased communication to cardiomyocytes (Cluster 03) from both AT2 cells (Cluster 00) and other cardiomyocytes (Cluster 03) pointed to a greater focus on vascular integrity and support (**Figure 5B**). The vaccine potentially triggers mechanisms that enhance the ability of the lung to manage blood flow and vascular stability during immune responses. The reduced signaling between AT2 cells, fibroblasts, and club cells reflected a shift away from ongoing tissue repair and maintenance, indicating that IN vaccination of L-DBF/ME had reduced the inflammatory burden (**Figure 5B**). This allowed a pulmonary focus on immune defense rather than tissue repair, optimizing the tissue environment for a more controlled and efficient immune response to the pathogen.

Furthermore, we found significant differences in signaling pathways between naïve and vaccinated groups (**Supplementary Figure S7**). In the naïve group, fibroblasts exhibited strong outgoing signaling through pathways such as Collagen, Laminin, Thbs (Thrombospondin), and Tenascin, all of which are involved in tissue structure and ECM interactions (**Supplementary Figure S7** Left) (33). These pathways also showed strong incoming signaling in the AT2 cell cluster, indicating significant ECM-related communication between these cell types. In contrast, cardiomyocytes demonstrated minimal outgoing and incoming activity in most of these pathways in the naïve group (**Supplementary Figure S7** Left).

After intranasal vaccination of L-DBF/ME, AT2 cells and cardiomyocytes showed increased outgoing signaling in Collagen, Laminin, Thbs, and Tenascin pathways. In contrast, fibroblasts showed reduced outgoing signaling in the same ECM-related pathways (**Supplementary Figure S7** Right). Fibroblasts and club cells exhibited reduced incoming signals in these structural pathways, suggesting less emphasis is placed on ECM interactions for these cells post-vaccination. Interestingly, Laminin showed increased incoming signaling in cardiomyocytes, while Thbs and Tenascin were reduced (**Supplementary Figure S7** Right). Moreover, fibroblasts showed increased outgoing signaling in ICAM, VCAM, and IGFBP pathways, which are important for cell adhesion and migration in immune responses (34, 35). For cardiomyocytes, both outgoing and incoming signaling in these immune-related pathways increased, reflecting the dual role of cardiomyocytes in supporting immune readiness while maintaining vascular integrity post-vaccination (**Supplementary Figure S7** Right).

## Discussion

Shigellosis remains a significant global public health issue, especially in regions with inadequate sanitation infrastructure (1). Climate change exacerbates its spread through extreme weather events (1). Vaccination to prevent shigellosis is sorely needed, particularly for vulnerable populations (4). Intranasal vaccination stimulates mucosal immunity that can target respiratory and gastrointestinal-associated lymphoid tissues, with the latter being where *Shigella* infections initiate (11). By promoting robust immune responses at mucosal surfaces, intranasal vaccines can enhance both local and systemic protection, providing a more effective defense against *Shigella* invasion in the gut (36). Our 'previous studies showed that the intranasal administration of three-dose regimen (one prime and two boosts) of L-DBF/ME formulation intranasally provides effective protection against lethal *Shigella* infection in a mouse pulmonary model (4). Administering a two-dose regimen (one prime and one boost) of L-DBF/ME allowed us to evaluate the minimum effective vaccination regimen while balancing efficacy and logistical feasibility (37). Reducing the number of doses could simplify vaccine administration and improve compliance while still achieving significant immune protection against *Shigella* infections (3). In this study, we demonstrate that two doses of the L-DBF/ME vaccine induced effective immune responses in mice, providing ≥50% protection against lethal *Shigella* infection. Intranasal L-DBF/ME elicited both humoral and cellular immune responses, including a primed Th17 response, as evidenced by the ability of lung immune cells to secrete IL-17A upon antigen stimulation, along with increased antibody production. Unlike findings from previous studies involving three vaccine doses, we did not observe a significant increase in IFN-γ levels upon antigen stimulation (4). This suggests that the two-dose regimen of L-DBF/ME vaccination might not fully stimulate the Th1 pathway, which may be critical for robust protection against *Shigella*. This would explain why the two vaccine doses don’t confer complete protection compared to the higher efficacy previously shown with three doses.

The spatial transcriptomic data presented here demonstrate that the two doses of intranasal L-DBF/ME successfully induce a coordinated immune defense at a mucosal site. In vaccinated mice, the localized upregulation of B-cell markers (*Cd19*, *Cd79a*) and T-cell markers (*Cd3e*, *Cd4*, *Cd8a*) showed strong adaptive immune system activation. When analyzing the outcomes of cell clusters, we found that B-cell activation markers were primarily found in fibroblasts and cardiomyocytes cluster-specific spots, suggesting that these cell types were important in facilitating B-cell activation or interactions in the lung environment post-vaccination (**Supplementary Table S2**) (38, 39). In contrast, T-cell markers were more broadly distributed across all cluster-specific spots, with high expression in spots corresponding to fibroblasts, indicating that fibroblasts were also vital in T-cell activation and immune regulation throughout the lung (**Supplementary Table S2**). This widespread expression of T-cell markers across various cluster-specific spots suggested that L-DBF/ME could induce a coordinated and well-distributed adaptive immune response, ensuring that multiple cell types were engaged in immune surveillance and activation against *Shigella* infection (38). Given the limited resolution of VST, it is possible that these signals in cluster-specific spots partly reflect ambient RNA from neighboring immune cells or transient paracrine signaling effects in the tissue microenvironment (40). Nonetheless, the concentration of immune markers in cluster-specific spots, particularly in these fibroblast-associated spots, suggested enhanced immune cell presence and communication in fibroblast regions, supporting the possibility that fibroblasts participate in immune interactions post-vaccination. Due to these limitations, further studies are needed to distinguish between direct expression by specific cell clusters and potential spatial proximity effects.

We also observed broad increases in the expression of *Fcgr1*, *Ly6i*, and *Prg2* across most clusters (**Figure 4**, **Supplementary Table S2**), further indicating the recruitment of macrophages and eosinophils, which are important in cytotoxic responses and pathogen clearance (41). Moreover, we found that L-DBF/ME primed lung immune cells for an IL-17 response upon antigen stimulation while maintaining immune homeostasis in the absence of infection (**Figure 1C**, **Supplementary Table S8A** left) (42). This is indicated by the lack of sustained upregulation at the transcriptomic level of *Il17a* and other neutrophil-related genes (*Cxcr2*, *Camp*, *Ly6g*) at 28 days after last vaccination (**Supplementary Table S8B**). Additionally, the observed down-regulation of *S100a8* and *S100a9* in vaccinated animals further supports the notion that inflammation was effectively regulated, preventing excessive neutrophil-driven responses while preserving immune readiness for pathogen challenge (31, 32). This suggests that L-DBF/ME not only boosts immune activation but also helps to control excessive inflammation, preventing potential tissue damage while maintaining a robust defense against the pathogen.

These outcomes indicate an increase in fibroblast numbers and changes in their communication patterns post-vaccination with L-DBF/ME. This suggests that fibroblasts, traditionally considered to be structural cells, may take on a somewhat specialized role in the lung immune microenvironment following intranasal vaccination of L-DBF/ME. Prior research has shown that reprogramming fibroblastic stromal cells (FSCs) in the lung is essential for maintaining protective memory CD8+ T cells and long-term immune protection via creating microenvironmental niches post-vaccination (43). We observed that the expression of *Cd8a*, a typical marker for CD8+ T cells, was exclusively found in those spots that also express the fibroblast cluster post-vaccination. These findings hint at a possible role for fibroblasts in immune-related processes following L-DBF/ME vaccination, though additional studies are required to confirm any direct reprogramming effect (43). Moreover, the reduced interaction between fibroblasts and other cell types following L-DBF/ME vaccination, along with decreased fibroblast-related self-interactions (typically involved in ECM production and tissue repair), suggests that fibroblasts have shifted away from their traditional role in maintaining tissue structure (17). These post-vaccination changes in signaling pathways further highlighted a shift in the functional roles of fibroblasts from ECM-related pathways to immune pathways like ICAM, VCAM, and Mif. While these observations imply that fibroblasts participate in immune interactions, further studies are needed to confirm whether these shifts are active in immune regulation or are secondary effects within the lung environment post-vaccination (17).

The presence of cardiomyocytes in pulmonary veins has been noted previously (30), but their potential role in immune regulation and vascular integrity within the lung environment remains unexplored. This study provides novel evidence suggesting that lung-associated cardiomyocytes may actively contribute to immune responses, offering a new perspective on cardiomyocyte function beyond the heart (18, 44). The detection of cardiomyocytes, typically found in heart tissue, in our lung samples was likely due to the proximity of our collection site near the main bronchi and the pulmonary veins, which carry oxygenated blood from the lungs to the heart (30). Following intranasal L-DBF/ME vaccination, lung-associated cardiomyocytes potentially contribute to vascular stability through enhanced ECM signaling, particularly for Laminin, while also being activated in immune regulation by influencing both outgoing and incoming immune signals (30). The increased ICAM and VCAM signaling observed post-vaccination suggested that cardiomyocytes facilitated immune cell adhesion and migration, supporting the recruitment of immune cells such as T cells and macrophages (30, 45). This modulation points to the potential for vaccines to leverage these pathways to induce a broader systemic immune response, with further investigation needed to confirm these functional roles. Additionally, the proximity of these cardiomyocytes to the pulmonary veins, which are directly connected to the circulatory system, further support the idea that immune responses initiated in the lungs can extend systemically (45). These findings suggest that L-DBF/ME triggers localized and systemic immune responses, underscoring its potential as a broad-spectrum protective vaccine, even in the gut. Further studies are needed to validate these observations and elucidate the mechanistic pathways through which cardiomyocytes influence pulmonary immune regulation and vascular dynamics.

In conclusion, two intranasal L-DBF/ME vaccination doses induced robust humoral and cellular immune responses in mice, offering moderate protection against lethal *Shigella* infection. While IFN-γ levels did not increase significantly upon antigen stimulation, the L-DBF/ME vaccine primed lung immune cells for a Th17 response, highlighting its potential for mucosal immunity. Additionally, beyond classical immune cell activation, intranasal L-DBF/ME appeared to influence fibroblasts and cardiomyocytes, potentially enhancing immune regulation and vascular integrity. This study provides new insights into the roles of non-immune cells, such as fibroblasts and cardiomyocytes, in regulating immunity and maintaining vascular stability in the lung. This highlights a potential novel function for cardiomyocytes in immune responses beyond their traditional role in cardiac physiology and shows how these cells contribute to immune defense following vaccination. Furthermore, the findings demonstrate the potential of intranasal vaccines to enhance mucosal immunity, offering improved long-term protection against Shigella and other mucosal pathogens.

## Data Availability

The data presented in this study are deposited in the Gene Expression Omnibus (GEO) repository under accession number GSE294222.

## References

[1] Lu T Das S Howlader DR Picking WD Picking WL. Shigella vaccines: the continuing unmet challenge. Int J Mol Sci. (2024) 25:4329. doi: 10.3390/ijms25084329 38673913 PMC11050647

[2] Giersing BK Isbrucker R Kaslow DC Cavaleri M Baylor N Maiga D. Clinical and regulatory development strategies for Shigella vaccines intended for children younger than 5 years in low-income and middle-income countries. Lancet Glob Health. (2023) 11:e1819–e26. doi: 10.1016/S2214-109X(23)00421-7 PMC10603611 37858591

[3] Barry EM Pasetti MF Sztein MB Fasano A Kotloff KL Levine MM. Progress and pitfalls in Shigella vaccine research. Nat Rev Gastroenterol Hepatol. (2013) 4:245–5. doi: 10.1038/nrgastro.2013.12 PMC3747556 23419287

[4] Lu T Raju M Howlader DR Dietz ZK Whittier SK Varisco DJ. Vaccination with a protective ipa protein-containing nanoemulsion differentially alters the transcriptomic profiles of young and elderly mice following shigella infection. Vaccines. (2024) 12:618. doi: 10.3390/vaccines12060618 38932347 PMC11209624

[5] Muthuramalingam M Whittier SK Picking WL Picking WD. The shigella type III secretion system: an overview from top to bottom. Microorganisms. (2021) 9:451. doi: 10.3390/microorganisms9020451 33671545 PMC7926512

[6] Martinez-Becerra FJ Chen X Dickenson NE Choudhari SP Harrison K Clements JD. Characterization of a novel fusion protein from IpaB and IpaD of *Shigella* spp. and its potential as a pan-*Shigella* vaccine. Infection Immun. (2013) 81:4470–7. doi: 10.1128/IAI.00859-13 PMC3837967 24060976

[7] Martinez-Becerra FJ Kissmann JM Diaz-McNair J Choudhari SP Quick AM Mellado-Sanchez G. Broadly protective *Shigella* vaccine based on type III secretion apparatus proteins. Infection Immun. (2012) 80:1222–31. doi: 10.1128/IAI.06174-11 PMC3294653 22202122

[8] Lu T Das S Howlader DR Zheng Q Siva Sai Kumar R Whittier SK. L-DBF elicits cross protection against different serotypes of Shigella spp. Front Trop Dis. (2021) 2. doi: 10.3389/fitd.2021.729731

[9] Lu T Das S Howlader DR Jain A Hu G Dietz ZK. Impact of the TLR4 agonist BECC438 on a novel vaccine formulation against Shigella spp. Front Immunol. (2023) 14:1194912. doi: 10.3389/fimmu.2023.1194912 37744341 PMC10512073

[10] Sellge G Magalhaes JG Konradt C Fritz JH Salgado-Pabon W Eberl G. Th17 cells are the dominant T cell subtype primed by Shigella flexneri mediating protective immunity. J Immunol. (2010) 184:2076–85. doi: 10.4049/jimmunol.0900978 20089698

[11] Lycke N. Recent progress in mucosal vaccine development: potential and limitations. Nat Rev Immunol. (2012) 12:592–605. doi: 10.1038/nri3251 22828912

[12] Dotiwala F Upadhyay AK. Next generation mucosal vaccine strategy for respiratory pathogens. Vaccines (Basel). (2023) 11:1585. doi: 10.3390/vaccines11101585 37896988 PMC10611113

[13] Jiang Y Hao S Chen X Cheng M Xu J Li C. Spatial transcriptome uncovers the mouse lung architectures and functions. Front Genet. (2022) 13:858808. doi: 10.3389/fgene.2022.858808 35391793 PMC8982079

[14] Williams CG Lee HJ Asatsuma T Vento-Tormo R Haque A. An introduction to spatial transcriptomics for biomedical research. Genome Med. (2022) 14:68. doi: 10.1186/s13073-022-01075-1,13 35761361 PMC9238181

[15] Su J Song Y Zhu Z Huang X Fan J Qiao J. Cell-cell communication: new insights and clinical implications. Signal Transduct Target Ther. (2024) 9:196. doi: 10.1038/s41392-024-01888-z 39107318 PMC11382761

[16] van de Verg LL Mallett CP Collins HH Larsen T Hammack C Hale TL. Antibody and cytokine responses in a mouse pulmonary model of Shigella flexneri serotype 2a infection. Infection Immun. (1995) 63:1947–54. doi: 10.1128/iai.63.5.1947-1954.1995 PMC173248 7729907

[17] Lee B Lee SH Shin K. Crosstalk between fibroblasts and T cells in immune networks. Front Immunol. (2022) 13:1103823. doi: 10.3389/fimmu.2022.1103823 36700220 PMC9868862

[18] McLendon JM Zhang X Matasic DS Kumar M Koval OM Grumbach IM. Knockout of sorbin and SH3 domain containing 2 (Sorbs2) in cardiomyocytes leads to dilated cardiomyopathy in mice. J Am Heart Assoc. (2022) 11:e025687. doi: 10.1161/JAHA.122.025687 35730644 PMC9333371

[19] Iyer V Cayatte C Guzman B Schneider-Ohrum K Matuszak R Snell A. Impact of formulation and particle size on stability and immunogenicity of oil-in-water emulsion adjuvants. Hum Vaccin Immunother. (2015) 11:1853–64. doi: 10.1080/21645515.2015.1046660 PMC4517459 26090563

[20] Jiang Y Li Y Cheng M Xu J Wei X Liu C. Protocol for acquiring high-quality fresh mouse lung spatial transcriptomics data. STAR Protoc. (2024) 5:102825. doi: 10.1016/j.xpro.2023.102825 38280199 PMC10840344

[21] Mesa AM Mao J Medrano TI Bivens NJ Jurkevich A Tuteja G. Spatial transcriptomics analysis of uterine gene expression in enhancer of zeste homolog 2 conditional knockout micedagger. Biol Reprod. (2021) 105:1126–39. doi: 10.1093/biolre/ioab147 PMC8599041 34344022

[22] Computing RFfS. R: A language and environment for statistical computing: RA Lang Environ Stat Comput. Vienna: R Foundation for Statistical Computing (2018).

[23] Hao Y Hao S Andersen-Nissen E Mauck WM 3rd Zheng S Butler A. Integrated analysis of multimodal single-cell data. Cell. (2021) 184:3573–87 e29. doi: 10.1016/j.cell.2021.04.048 34062119 PMC8238499

[24] Hafemeister C Satija R. Normalization and variance stabilization of single-cell RNA-seq data using regularized negative binomial regression. Genome Biol. (2019) 20:296. doi: 10.1186/s13059-019-1874-1 31870423 PMC6927181

[25] Jin S Guerrero-Juarez CF Zhang L Chang I Ramos R Kuan CH. Inference and analysis of cell-cell communication using CellChat. Nat Commun. (2021) 12:1088. doi: 10.1038/s41467-021-21246-9 33597522 PMC7889871

[26] Mitchell PS Roncaioli JL Turcotte EA Goers L Chavez RA Lee AY. NAIP-NLRC4-deficient mice are susceptible to shigellosis. Elife. (2020) 9:e59022. doi: 10.7554/eLife.59022 33074100 PMC7595732

[27] Akinrinmade OA Chetty S Daramola AK Islam MU Thepen T Barth S. CD64: an attractive immunotherapeutic target for M1-type macrophage mediated chronic inflammatory diseases. Biomedicines. (2017) 5:56. doi: 10.3390/biomedicines5030056 28895912 PMC5618314

[28] Fulkerson PC Rothenberg ME. Eosinophil development, disease involvement, and therapeutic suppression. Adv Immunol. (2018) 138:1–34. doi: 10.1016/bs.ai.2018.03.001 29731004

[29] Jang DI Lee AH Shin HY Song HR Park JH Kang TB. The role of tumor necrosis factor alpha (TNF-alpha) in autoimmune disease and current TNF-alpha inhibitors in therapeutics. Int J Mol Sci. (2021) 22:2719. doi: 10.3390/ijms22052719 33800290 PMC7962638

[30] Folmsbee SS Gottardi CJ. Cardiomyocytes of the heart and pulmonary veins: novel contributors to asthma? Am J Respir Cell Mol Biol. (2017) 57:512–8. doi: 10.1165/rcmb.2016-0261TR PMC5705903 28481622

[31] Wang S Song R Wang Z Jing Z Wang S Ma J. S100A8/A9 in inflammation. Front Immunol. (2018) 9:1298. doi: 10.3389/fimmu.2018.01298 29942307 PMC6004386

[32] Sprenkeler EGG Zandstra J van Kleef ND Goetschalckx I Verstegen B Aarts CEM. S100A8/A9 is a marker for the release of neutrophil extracellular traps and induces neutrophil activation. Cells. (2022) 11:236. doi: 10.3390/cells11020236 35053354 PMC8773660

[33] Tomko LA Hill RC Barrett A Szulczewski JM Conklin MW Eliceiri KW. Targeted matrisome analysis identifies thrombospondin-2 and tenascin-C in aligned collagen stroma from invasive breast carcinoma. Sci Rep. (2018) 8:12941. doi: 10.1038/s41598-018-31126-w 30154546 PMC6113240

[34] Harjunpaa H Llort Asens M Guenther C Fagerholm SC. Cell adhesion molecules and their roles and regulation in the immune and tumor microenvironment. Front Immunol. (2019) 10:1078. doi: 10.3389/fimmu.2019.01078 31231358 PMC6558418

[35] Baxter RC. Signaling pathways of the insulin-like growth factor binding proteins. Endocr Rev. (2023) 44:753–78. doi: 10.1210/endrev/bnad008 PMC10502586 36974712

[36] Lavelle EC Ward RW. Mucosal vaccines - fortifying the frontiers. Nat Rev Immunol. (2022) 22:236–50. doi: 10.1038/s41577-021-00583-2 PMC8312369 34312520

[37] Dogra P Schiavone C Wang Z Ruiz-Ramirez J Caserta S Staquicini DI. A modeling-based approach to optimize COVID-19 vaccine dosing schedules for improved protection. JCI Insight. (2023) 8:e169860. doi: 10.1172/jci.insight.169860 37227783 PMC10371350

[38] Gerasimova EV Tabakov DV Gerasimova DA Popkova TV. Activation markers on B and T cells and immune checkpoints in autoimmune rheumatic diseases. Int J Mol Sci. (2022) 23:8656. doi: 10.3390/ijms23158656 35955790 PMC9368764

[39] Bieberich F Vazquez-Lombardi R Yermanos A Ehling RA Mason DM Wagner B. A single-cell atlas of lymphocyte adaptive immune repertoires and transcriptomes reveals age-related differences in convalescent COVID-19 patients. Front Immunol. (2021) 12:701085. doi: 10.3389/fimmu.2021.701085 34322127 PMC8312723

[40] Lee J Yoo M Choi J. Recent advances in spatially resolved transcriptomics: challenges and opportunities. BMB Rep. (2022) 55:113–24. doi: 10.5483/BMBRep.2022.55.3.014 PMC8972138 35168703

[41] Mantovani A Allavena P Marchesi F Garlanda C. Macrophages as tools and targets in cancer therapy. Nat Rev Drug Discovery. (2022) 21:799–820. doi: 10.1038/s41573-022-00520-5 35974096 PMC9380983

[42] Zenobia C Hajishengallis G. Basic biology and role of interleukin-17 in immunity and inflammation. Periodontol 2000. (2015) 69:142–59. doi: 10.1111/prd.2015.69.issue-1 PMC4530463 26252407

[43] Cupovic J Ring SS Onder L Colston JM Lutge M Cheng HW. Adenovirus vector vaccination reprograms pulmonary fibroblastic niches to support protective inflating memory CD8(+) T cells. Nat Immunol. (2021) 22:1042–51. doi: 10.1038/s41590-021-00969-3 PMC7611414 34267375

[44] Szibor M Poling J Warnecke H Kubin T Braun T. Remodeling and dedifferentiation of adult cardiomyocytes during disease and regeneration. Cell Mol Life Sci. (2014) 71:1907–16. doi: 10.1007/s00018-013-1535-6 PMC11113405 24322910

[45] Klaourakis K Vieira JM Riley PR. The evolving cardiac lymphatic vasculature in development, repair and regeneration. Nat Rev Cardiol. (2021) 18:368–79. doi: 10.1038/s41569-020-00489-x PMC7812989 33462421

