# Supplementary material for: Spatial visualization provides insight into immune modulation by an L-DBF vaccine formulation against Shigella
Source: Front Immunol. 2025 Apr 23;16:1577040. doi: 10.3389/fimmu.2025.1577040 (PMC12056741; doi:10.3389/fimmu.2025.1577040)
Supplement: Supplementary file 1 [file DataSheet1.pdf]

Supplemental Information for . . .

Spatial Visualization Provides Insight into Immune Modulation by an L-DBF Vaccine Formulation Against *Shigella*

Ti Lu[1*], Skyler T. Kramer[2], Mary A. York[2], Mst Nusrat Zahan[1], Debaki R. Howlader[1], Zackary K. Dietz[1], Sean K. Whittier[1], Nathan J. Bivens[3], Alexander Jurkevich[4], Lyndon M. Coghill[1, 2], William D. Picking[1], and Wendy L. Picking[1*]

[1]Bond Life Sciences Center and Department of Veterinary Pathobiology, University of Missouri, Columbia, MO 65211, USA; [2]Bioinformatics and Analytic Core, University of Missouri Columbia MO 65211; [3]Genomics Technology Core, University of Missouri, Columbia, MO, USA; [4]Advanced Light Microscopy Core, University of Missouri, Columbia, MO, USA.

*Co-corresponding Authors: Wendy L Picking, wendy.picking@missouri.edu; Ti Lu, tilu@missouri.edu.

Supplemental Table S1. Acronyms used in this paper.

T3SS	Type III secretion system
DBF	IpaD-IpaB fusion protein
dmLT	Double-mutant heat-labile enterotoxin
LTA1	A1 moiety of the active subunit of dmLT
L-DBF	LTA1 fusion with DBF
ETEC	Enterotoxigenic Escherichia coli
IN	Intranasal
LDAO	Lauryl-dimethylamine oxide
ME	MedImmune emulsion
MOPS	3-(N-morpholino)propanesulfonic acid
IPTG	Isopropyl β-D-1-thiogalactopyranoside
IMAC	Immobilized Metal Affinity Chromatography
EU	Endotoxin Unit
BECC	Bacterial Engineering Combinatorial Chemistry
OPD	O-phenylenediamine dihydrochloride
OCT	Optimal Cutting Temperature
VST	Visual-spatial transcriptomics

Supplemental Table S2. Differential expression analyses (DEAs) of key genes that compare naïve vs vaccinated samples within each cluster at the 5% levels of threshold.

Cluster	Gene	P_Val	Avg_Log2fc	Naïve	Vaccinated	P_Val_Adj
Cluster 0	S100a8	3.27E-146	1.5909524	0.274	0.103	1.73E-144
Cluster 0	S100a9	1.76E-188	1.72628028	0.3	0.1	1.35E-186
Cluster 0	Cd4	1.77E-73	-2.6650437	0.012	0.079	4.50E-72
Cluster 0	Cd3e	3.61E-49	-1.877452	0.024	0.082	6.22E-48
Cluster 0	Fcgr1	4.12E-45	-1.957048854	0.018	0.069	6.32E-44
Cluster 1	S100a8	0.00013848	0.74719526	0.17	0.107	0.00033167
Cluster 1	S100a9	1.49E-06	0.91747812	0.185	0.106	4.55E-06
Cluster 1	Cd4	6.69E-14	-1.9738902	0.046	0.171	3.61E-13
Cluster 1	Cd3e	2.18E-26	-2.8642692	0.029	0.224	1.99E-25
Cluster 1	Cd8a	3.31E-06	-2.2188943	0.012	0.06	9.74E-06
Cluster 1	Cd79a	9.85E-32	-2.7819176	0.08	0.329	1.04E-30
Cluster 1	Cd19	2.45E-06	-1.987421181	0.031	0.09	7.32E-06
Cluster 1	Fcgr1	3.60E-06	-1.604683597	0.026	0.082	1.05E-05
Cluster 2	S100a8	1.96E-05	1.6428685	0.091	0.039	5.27E-05
Cluster 2	S100a9	6.49E-07	1.60934517	0.113	0.046	2.05E-06
Cluster 2	Cd3e	1.55E-08	-2.2411044	0.015	0.073	5.82E-08
Cluster 3	S100a8	5.83E-05	1.30596238	0.167	0.054	0.00014662
Cluster 3	S100a9	2.38E-05	1.67752125	0.179	0.059	6.30E-05
Cluster 3	Cd4	1.04E-11	-3.4145151	0.012	0.117	4.91E-11
Cluster 3	Cd3e	3.40E-11	-3.1414966	0.017	0.127	1.56E-10
Cluster 3	Cd79a	5.02E-17	-2.9435572	0.04	0.229	3.19E-16
Cluster 3	Cd19	0.002113245	-1.682064974	0.015	0.054	0.004026243
Cluster 3	Fcgr1	0.000376399	-1.656069766	0.018	0.068	0.000837409

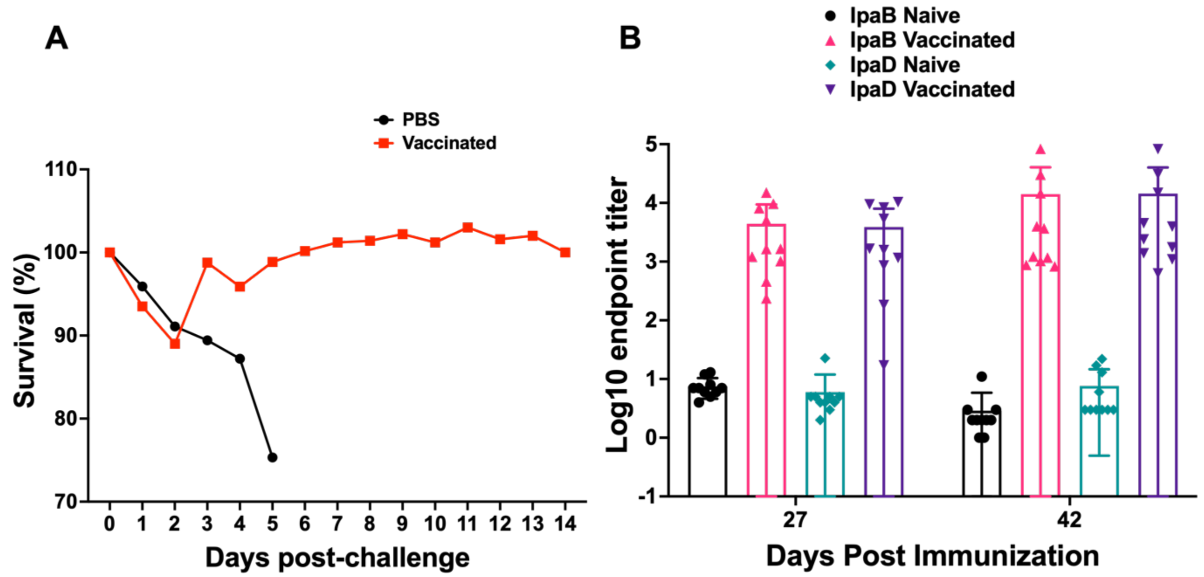

Supplemental Figure S1. (A) Weight loss for the mice post-infection. The timeline (x-axis) shows the percentage of weight (y- axis) change over time. (B) The kinetics of serum IgG titers from naïve (black/green) and vaccinated (red/purple) mice for days 27 and 42 is shown as the titers for anti-IpaB (circle/up triangle) or anti-IpaD (square/ down triangle) based on ELISA. The individual titers are represented as EU mL^{-1}. Each point represents the mean of each group (n = 10/group).

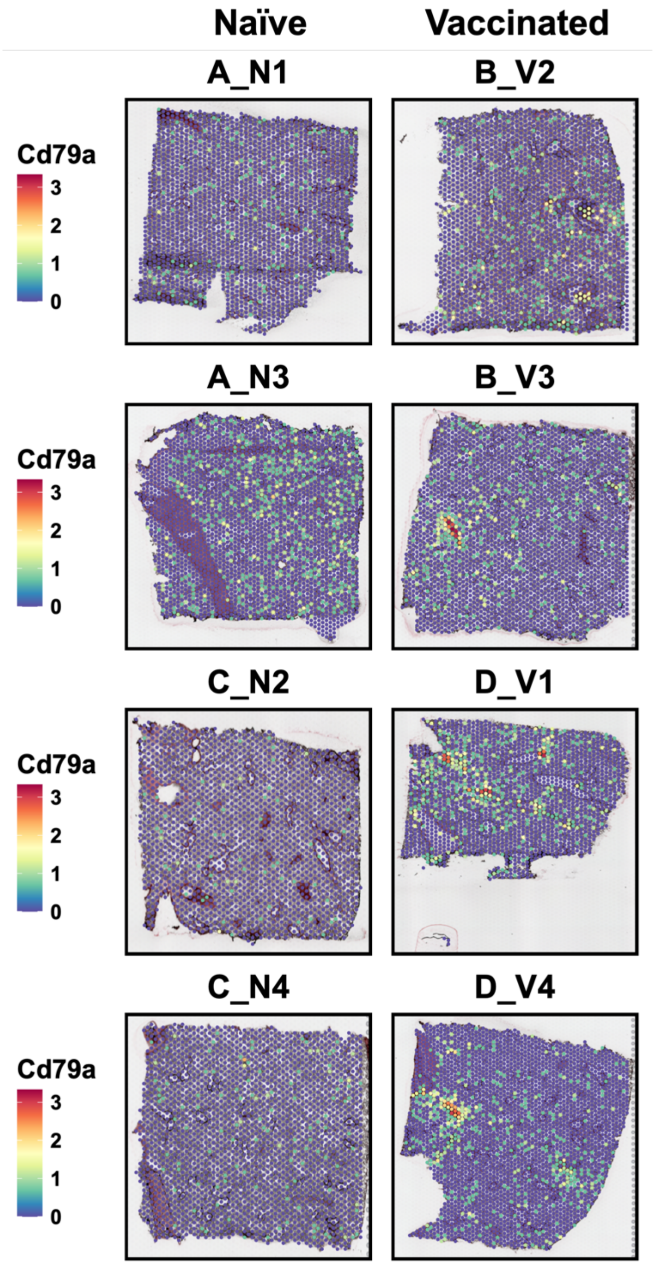

Supplemental Figure S2. Spatial transcriptomic maps of lung sections, showing the distribution and expression levels of the markers Cd79a. Each panel represents a different sample, labeled as A_N1, B_V2, A_N3, B_V3, C_N2, D_V1, C_N4, and D_V4. The color scale on each map indicates the expression level of the markers, with red and yellow denote higher expression levels, while blue represents lower levels. The labels "N" and "V" suggest naïve and vaccinated groups, respectively, with numbers indicating specific sample identifiers.

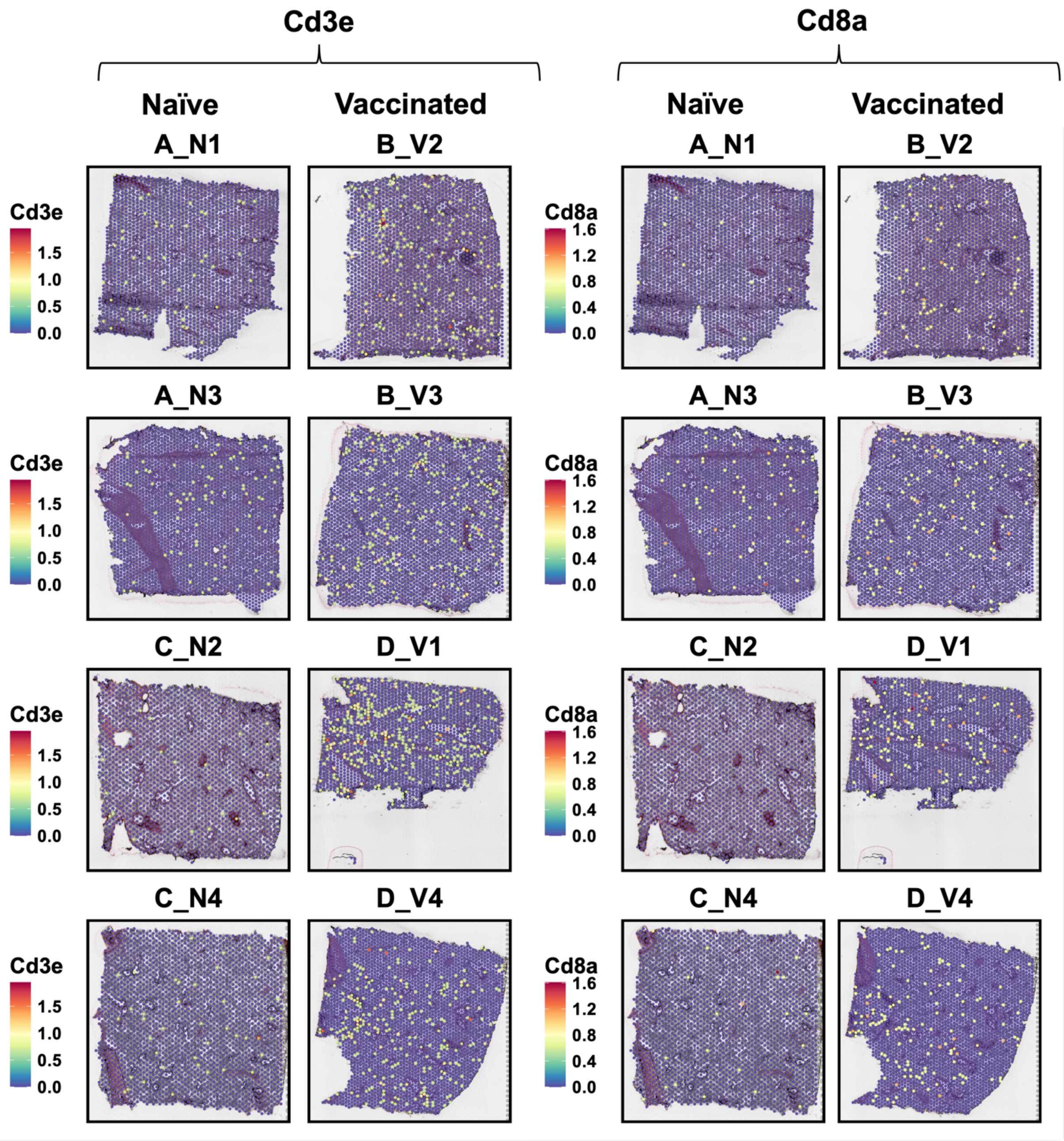

Supplemental Figure S3. Spatial transcriptomic maps of lung sections, showing the distribution and expression levels of the markers Cd3e (left) and Cd8a (right). Each panel represents a different sample, labeled as A_N1, B_V2, A_N3, B_V3, C_N2, D_V1, C_N4, and D_V4. The color scale on each map indicates the expression level of the markers, with red and yellow denote higher expression levels, while blue represents lower levels. The labels "N" and "V" suggest naïve and vaccinated groups, respectively, with numbers indicating specific sample identifiers.

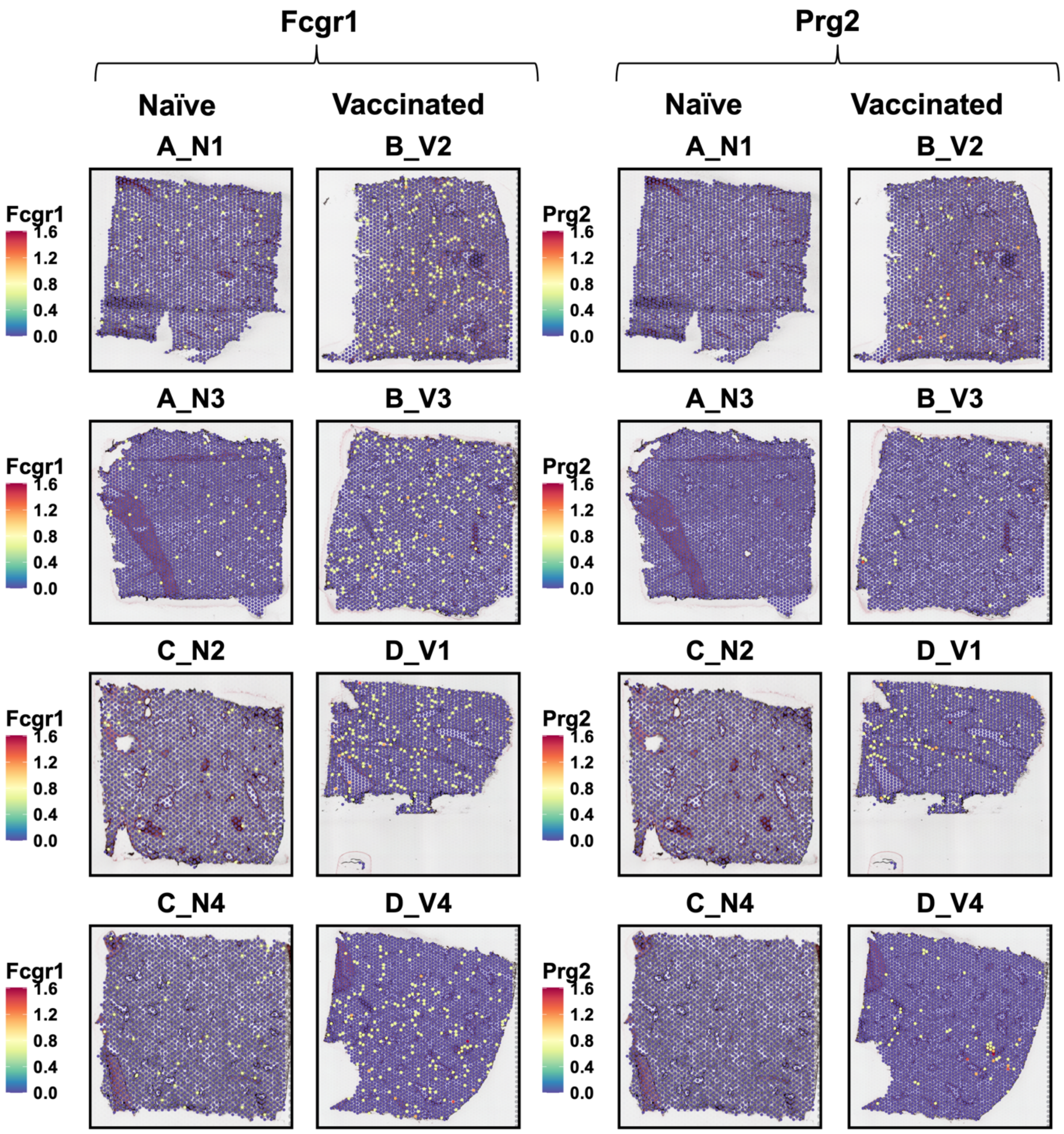

Supplemental Figure S4. Spatial transcriptomic maps of lung sections, showing the distribution and expression levels of the markers Fcgr1 (left) and Prg2 (right). Each panel represents a different sample, labeled as A_N1, B_V2, A_N3, B_V3, C_N2, D_V1, C_N4, and D_V4. The color scale on each map indicates the expression level of the markers, with red and yellow denote higher expression levels, while blue represents lower levels. The labels "N" and "V" suggest naïve and vaccinated groups, respectively, with numbers indicating specific sample identifiers.

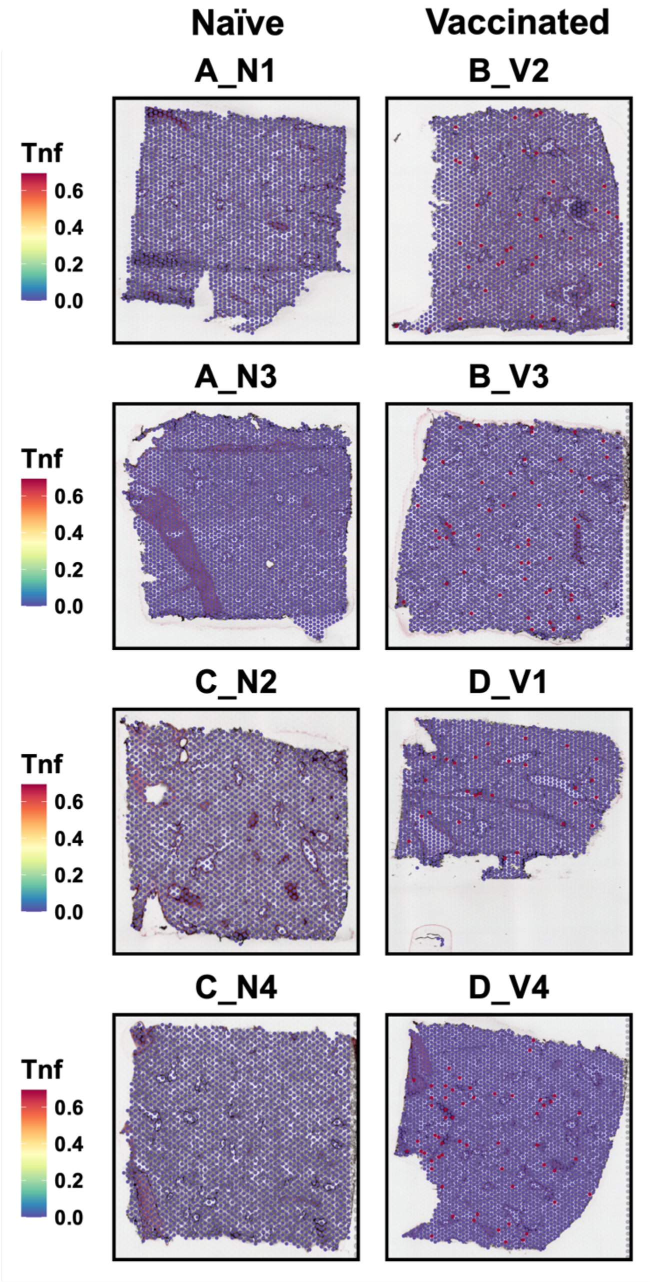

Supplemental Figure S5. Spatial transcriptomic maps of lung sections, showing the distribution and expression levels of the markers Tnf. Each panel represents a different sample, labeled as A_N1, B_V2, A_N3, B_V3, C_N2, D_V1, C_N4, and D_V4. The color scale on each map indicates the expression level of the markers, with red and yellow denote higher expression levels, while blue represents lower levels. The labels "N" and "V" suggest naïve and vaccinated groups, respectively, with numbers indicating specific sample identifiers.

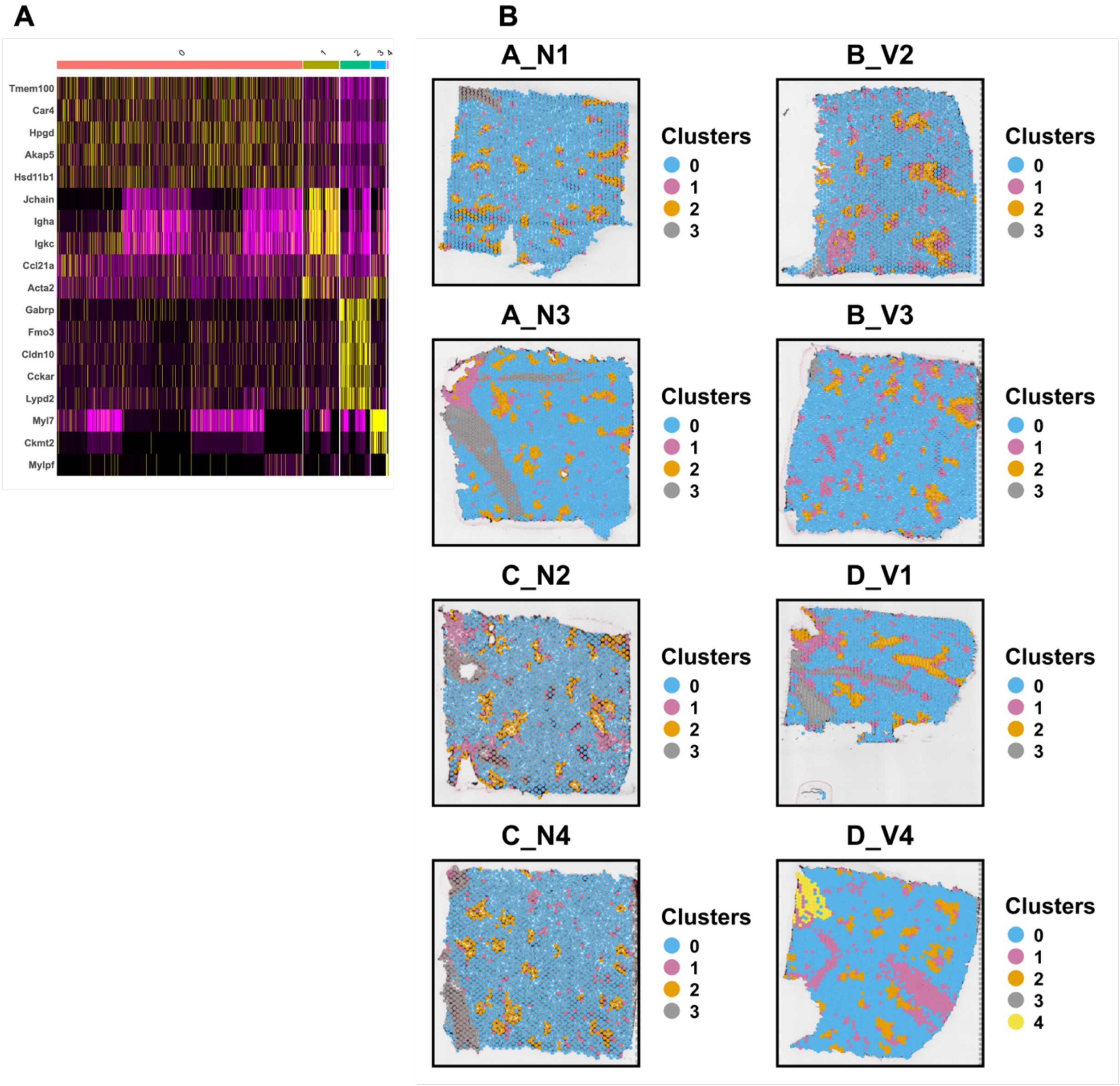

Supplemental Figure S6. (A) Heatmap displays the expression profiles of various genes across identified cell clusters, with color intensity reflecting expression levels, ranging from low (purple) to high (yellow). (B) Adjacent maps illustrate the spatial distribution of these clusters within each section, labeled as clusters 0 through 4. Each panel represents a different sample, labeled as A_N1, B_V2, A_N3, B_V3, C_N2, D_V1, C_N4, and D_V4. Each color in the maps corresponds to a different cluster, helping to visualize the cellular heterogeneity and distribution patterns in the lung tissues.

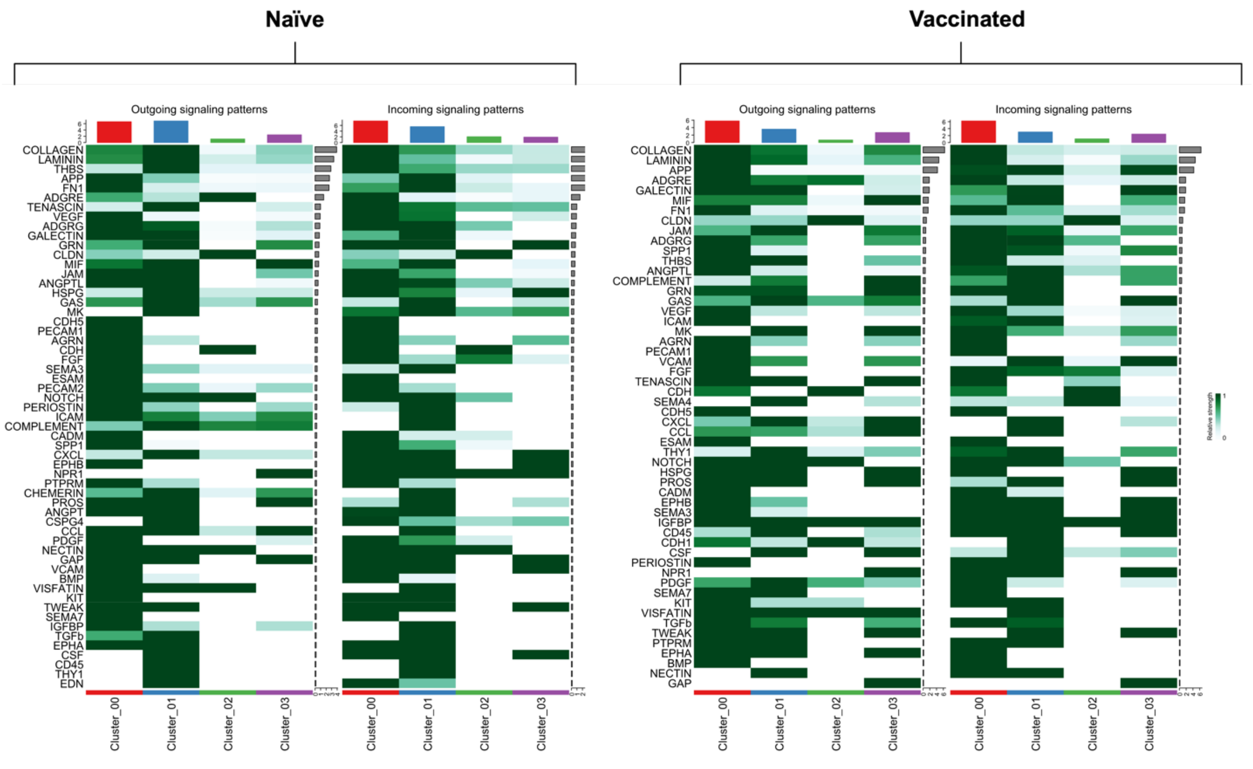

Supplemental Figure S7. Heatmaps display outgoing and incoming signaling patterns in different cell clusters (Cluster 0, Cluster 1, Cluster 2, Cluster 3) identified from a spatial transcriptomics analysis. Each column represents a specific cluster, while rows correspond to various signaling molecules. Shades of green indicate the relative expression level of each signaling molecule, with darker shades representing higher expression levels. The top bar plots are the total values of each column.

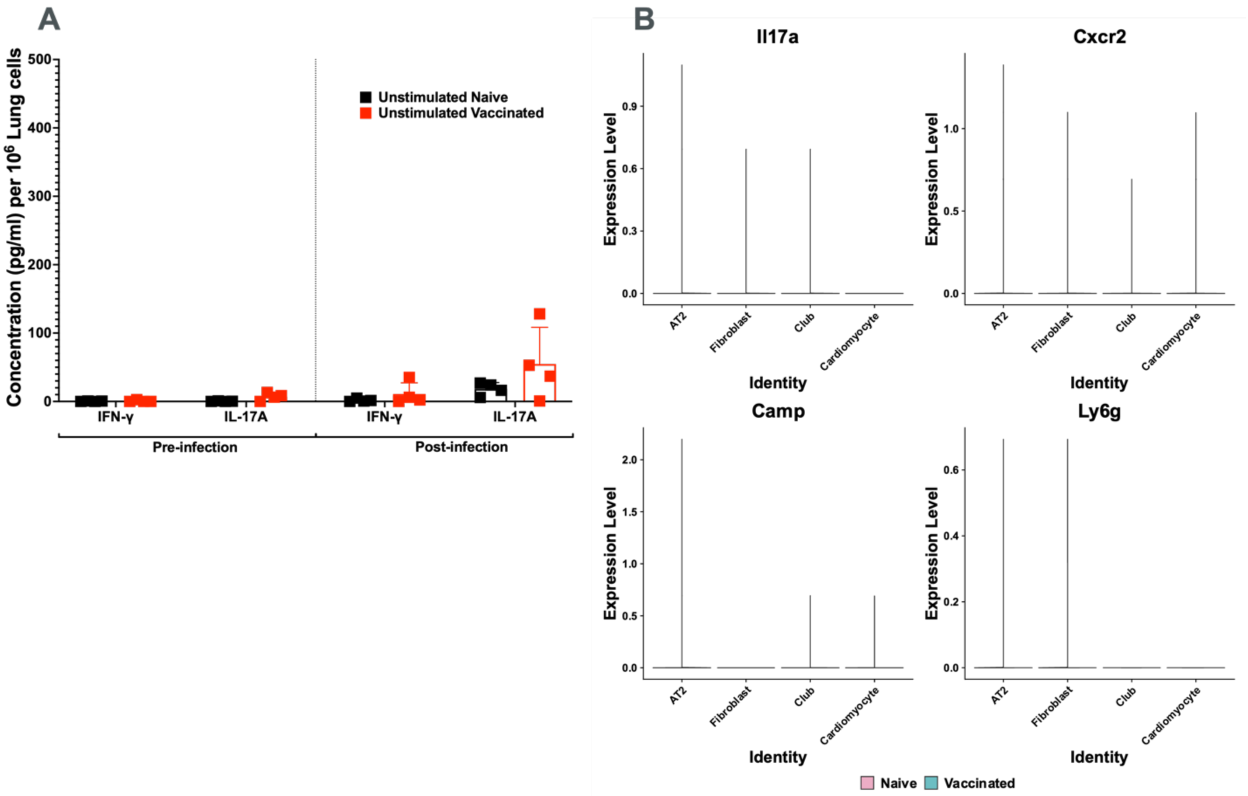

Supplemental Figure S8. (A) Cytokine levels from pre- and post-infection in naïve (black) and vaccinated (red) groups were determined by Meso Scale Discovery analysis as per the manufacturer's specifications. They are presented here as pg/ml/10^6 lung cells. Secretion of different cytokines was noted as a response to unstimulating (PBS). Data were plotted as actual values from individuals ± SD (n = 4) in each group. (B) Violin plots are shown comparing the expression levels of selected immune-related genes across different cell clusters (Alveolar type 2 (AT2) Cells; Fibroblasts; Club cells; Cardiomyocytes) identities in naïve and vaccinated groups. Each plot represents a different gene, including *Il17a, Cxcr2, Camp, Ly6g*. The x-axis labels cell cluster identities, and the y-axis represents expression levels. Pink represents the naïve group, while blue indicates the vaccinated group.
